# Pneumococcal Infection among Children before Introduction of 13-Valent Pneumococcal Conjugate Vaccine, Cambodia

**DOI:** 10.3201/eid2111.150914

**Published:** 2015-11

**Authors:** Paul Turner, Claudia Turner, Kuong Suy, Sona Soeng, Sokeng Ly, Thyl Miliya, David Goldblatt, Nicholas P.J. Day

**Affiliations:** Cambodia Oxford Medical Research Unit, Siem Reap, Cambodia (P. Turner, C. Turner, K. Suy);; Mahidol University, Bangkok, Thailand (P. Turner, C. Turner, N.P.J. Day);; University of Oxford, Oxford, UK (P. Turner. C. Turner, N.P.J. Day);; Angkor Hospital for Children, Siem Reap (S. Soeng, S. Ly, T. Miliya);; University College London, London, UK (D. Goldblatt)

**Keywords:** *Streptococcus pneumoniae*, infection, colonization, vaccine, children, bacteria, 13-valent pneumococcal conjugate vaccine, PVC13, Cambodia

## Abstract

Vaccination of children with pneumococcal conjugate vaccine (PCV13) was initiated in Cambodia in 2015. To determine baseline data, we collected samples from children in 2013 and 2014. PCV13 serotypes accounted for 62.7% of colonizing organisms in outpatients and 88.4% of invasive pneumococci overall; multidrug resistance was common. Thus, effectiveness of vaccination should be high.

Infection with *Streptococcus pneumoniae* remains a substantial cause of death among children (*1*). In high-income countries, introduction of pneumococcal conjugate vaccine (PCV) has substantially decreased incidence of invasive pneumococcal disease (IPD) (*2*). Data for PCV effect in low-income countries are less robust (*2*). We therefore studied the characteristics of pneumococci responsible for colonization and invasive disease among children in Cambodia before the early 2015 introduction of 13-valent PCV (PCV13).

## The Study

The study was conducted at Angkor Hospital for Children, Siem Reap, Cambodia. Before enrollment of a child, written consent was obtained from the parent/guardian. Ethical approval was granted by the hospital institutional review board and the Oxford Tropical Research Ethics Committee. For the colonization study, which was conducted in January (cool/dry season) and August (hot/wet season) 2014, colonization surveys were conducted in the outpatient department. Nasopharyngeal swab samples were collected from children 1 month to 15 years of age who had minor illnesses, excluding nonsevere pneumonia, not requiring hospital admission. Children were eligible for enrollment 1 time per survey. For the invasive disease study, which was conducted during August 1, 2013–July 31, 2014, samples were collected from hospitalized children 1 month to 15 years of age who met World Health Organization (WHO) clinical case definitions for pneumonia, meningitis, or sepsis (*3*). Children readmitted within 14 days were excluded from reenrollment. Samples were processed according to the WHO pneumococcal colonization detection protocol (*4*). Pneumococci were confirmed by optochin susceptibility and/or bile solubility and were serotyped by latex agglutination (*5*). Antimicrobial drug susceptibilities were determined according to Clinical and Laboratory Standards Institute guidelines (*6*). Serotype and antimicrobial drug susceptibilities were also determined for all invasive pneumococcal isolates cultured from patients during January 1, 2013–December 1, 2014. Pneumococci were grouped into vaccine serotypes (PCV13: 1, 3, 4, 5, 6A, 6B, 7F, 9V, 14, 18C, 19F, 19A, 23F), nonvaccine serotypes (all others), and nontypeable isolates. Multidrug resistance was defined as resistance to >3 agents (Technical Appendix) (*7*).

**Figure 1:**
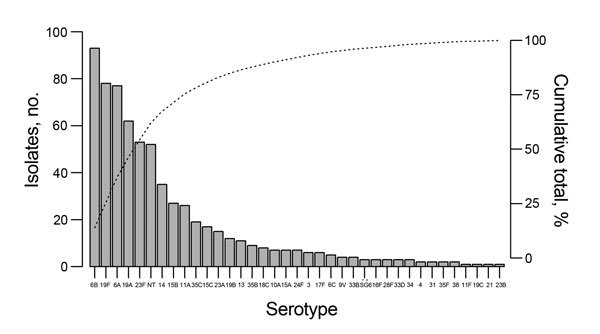
Serotype distribution of 667 pneumococcal isolates cultured from nasopharyngeal swab samples collected from 974 outpatients 1 month–15 years of age, at Angkor Hospital for Children, Cambodia, Siem Reap, January and August 2014. Bars indicate number of isolates; dotted line indicates cumulative total percentage of isolates.

The outpatient colonization study included 974 children (Table 1; Technical Appendix Figure 1). None were known to be HIV infected. Pneumococcal colonization was detected in 601 (61.7%) of children (Technical Appendix Table 1). Colonization prevalence declined with age: 78.6% (206/262) in those 1–11 months, 61.9% (284/459) in those 12–59 months, and 43.9% (111/253) in those ≥5 years of age. The proportion colonized were 75.2% (342/455) in the cool/dry season and 49.9% (259/519) in the hot/wet season (p<0.001). The adjusted odds ratio for colonization in the hot/wet season was 0.38 (95% CI 0.28–0.51, p<0.001) after controlling for age, household size, cohabitation with other young children, current upper respiratory tract symptoms, and recent antimicrobial use. A total of 667 pneumococci were isolated (Figure 1). Among 601 colonized children, >1 serotype was identified in 11.0% (66/601). PCV13 serotypes accounted for 62.7% (418/667), nonvaccine serotypes for 29.5% (197/667), and nontypeable isolates for 7.8% (52/667) of isolates. The proportion of children colonized by PCV13 serotypes was greater among those <5 years of age (70.2% [344/490]) than among older children (48.6% [54/111]); p<0.001; whereas the opposite was true for colonization with nonvaccine serotypes (27.8% [136/490] vs. 48.6% [54/111]; p<0.001). Colonization with nontypeable isolates did not vary by age (data not shown). Overall, 68.8% (459/667) of pneumococci were multidrug resistant: 85.4% of PCV13 isolates, 50.0% of nontypeable isolates, and 38.6% of nonvaccine serotypes (p<0.001). Among colonized children, multidrug-resistant pneumococci were more commonly cultured from children <5 years of age (75.1% [368/490]) than from older children (53.2% [59/111]); p<0.001.

**Table 1:** Characteristics of 974 children enrolled in outpatient pneumococcal colonization surveys at Angkor Hospital for Children, Siem Reap, Cambodia, January and August 2014*

Characteristic	Overall	January 2014	August 2014	p value†
Total no. enrolled	974	455	519	
Age, median (IQR)	2.5 (0.9–5.1)	1.9 (0.9–4.1)	2.9 (1.1–6.0)	<0.001
Age category, no. (%)				
1–11 mo	262 (26.9)	142 (31.2)	120 (23.1)	0.005
12–59 mo	459 (47.1)	231 (50.8)	228 (43.9)	0.03
5–15 y	253 (26.0)	82 (18.0)	171 (33.0)	<0.001
Male sex, no. (%)	499 (51.2)	241 (53.0)	258 (49.7)	0.3
Reason for outpatient visit, no. (%)				
Upper respiratory tract infection	794 (81.5)	394 (86.6)	400 (77.1)	<0.001
Gastroenteritis	92 (9.5)	47 (10.3)	45 (8.7)	0.4
Other	88 (9.0)	14 (3.1)	74 (14.2)	<0.001
Antimicrobial drug use in preceding month, no. (%)‡	453/967 (46.8)	149/453 (32.9)	175/514 (34.0)	0.7
Household size, median (IQR)	5 (4–6)	5 (4–7)	5 (4–6)	0.01
Other children <5 y of age in household, no. (%)	841/973 (86.4)	422/454 (93.0)	419 (80.7)	<0.001
Attendance at school or daycare, no. (%)	293/973 (30.1)	105/454 (23.1)	188 (36.2)	<0.001

*Weather conditions were cool and dry in January and hot and wet in August. Where data were missing, an alternate denominator is included in the affected cell. IQR, interquartile range. †For proportions, comparisons were made by using the χ^2^ test. For continuous variables, comparisons were made by using the Wilcoxon rank-sum test. ‡Includes definite and possible (unknown systemic medication) consumption in the community before outpatient visit.

**Figure 2:**
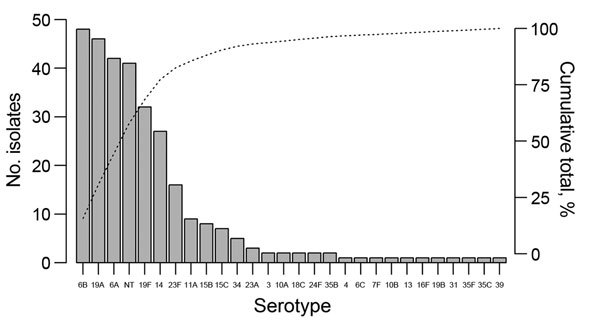
Serotype distribution of 305 pneumococcal isolates cultured from nasopharyngeal swab samples collected from 1,008 hospitalized patients 1 month–15 years of age at Angkor Hospital for Children, Siem Reap, Cambodia, August 2013–July 2014. Bars indicate number of isolates; dotted line indicates cumulative total percentage of isolates.

From August 1, 2013, through July 1, 2014, a total of 2,613 cases of medical admissions were screened; of these, 1,009 were included in the analysis (Technical Appendix Figure 1). Median patient age at admission was 1.2 years (interquartile range 0.5–2.4), 56.5% (570/1,009) of patients were male, and 1.4% (14/1,006) were HIV positive. Most cases met the WHO category of severe pneumonia (Technical Appendix Table 2). Pneumococcal colonization was identified in 29.1% (293/1,008) of children from whom a swab sample was obtained (Technical Appendix Table 3). Colonization was less frequent in those who had received >1 dose of an antimicrobial drug (most frequently ceftriaxone) in hospital before the swab sample collection (23.8% [187/785]) than among those who had not (48.5% [95/196]); p<0.001. Colonization was identified in 31.3% (175/559) of children during the dry seasons (hot: March–May; cool: November–February) and in 26.3% (118/449) during the wet season (June–October); p = 0.08. A total of 305 pneumococci were isolated, comprising 27 serotypes plus nontypeable isolates (Figure 2). PCV13 serotypes accounted for 71.1% (217/305) of isolates, nonvaccine serotypes for 15.4% (47/305), and nontypeable isolates for 13.4% (41/305). Multidrug resistance was found in 79.3% (242/305) of isolates.

During 2013–2014, a total of 43 cases of IPD were culture proven (online Technical Appendix). Median patient age was 2.5 years (interquartile range 1.4–8.6). Overall, PCV13 serotypes accounted for 38 (88.4%, 95% CI 74.9–96.1) infections (Table 2). Multidrug resistance was identified in 55.8% (24/43): 22/38 (57.9%) of PCV13 serotypes and 2 (40.0%) of 5 nonvaccine serotypes; p = 0.6. Full resistance profiles are provided in Technical Appendix Table 4.

**Table 2:** Serotypes of invasive pneumococcal isolates from hospitalized children, Angkor Hospital for Children, Siem Reap, Cambodia, 2013–2014*

Specimen type	No.	PCV13 serotype, no. (%)	Serotypes, (no.)
Blood	35	31 (88.6)	1 (10), 6B (9), 14 (4), 23F (3), 6A (2), 12F† (1), 16F† (1), 19F (1), 19A (1), 18C (1), 28F† (1), nontypeable† (1)
Cerebrospinal fluid‡	3	3 (100)	1 (1), 6B (1), 19A (1)
Pleural fluid‡	4	4 (100)	1 (2), 5 (1), 19A (1)
Vitreous fluid	1	0 (0)	Nontypeable (1)†
Total	43	38 (88.4)	1 (13), 6B (10), 14 (4), 19A (3), 23F (3), 6A (2), NT† (2), 5 (1), 12F† (1), 16F† (1), 19F (1), 18C (1), 28F† (1)

*PCV13, 13-valent pneumococcal conjugate vaccine. †Non-PCV13 serotypes. ‡Identical pneumococci were isolated from pleural fluid for 3 patients and from cerebrospinal fluid for 2 patients.

## Conclusions

This study highlights the high potential for reduction of IPD among children after introduction of PCV13 in Cambodia; 88.4% (95% CI 74.9–96.1) of invasive isolates from this 1 surveillance site were serotypes covered by the vaccine. Vaccination should result in decreased drug-resistant pneumococcal infections, although the substantial reservoir of resistance in nonvaccine type and nontypeable pneumococci will probably erode any reduction over time (*8**–**10*).

Colonization was high among outpatients and similar to that in other Southeast Asia locations (*5**,**11*). Multidrug resistance was common, probably the result of poor regulation of antimicrobial drug use in Cambodia (*12*); 72.1% of colonizing isolates and 55.8% of invasive isolates were multidrug resistant. For comparison, a recent study of children in Thailand found 31.6% of colonizing pneumococci to be multidrug resistant (*13*).

The range of serotypes detected in the colonization study was broad but slightly more restricted than that detected in other low-income country studies. In a longitudinal colonization study of refugee infants on the Thailand–Myanmar border, 67 serotypes were identified (*5*). This finding may reflect the high prevalence of antimicrobial drug use in the community, which would reduce the colonization prevalence of less resistant nonvaccine serotypes. However, the identification of several serotypes emerging as causes of IPD in South Africa, the United Kingdom, and the United States after introduction of PCV13 (e.g., serotypes 15A, 15B/C, 23B, 24F; which accounted for 7.8% of colonizing pneumococci in our study) is noteworthy, indicating the need for close monitoring for changes in colonization and IPD serotype distribution after PCV13 introduction (*7**,**14**,**15*).

The study has several limitations. The absolute number of IPD cases was small, and it was not possible to calculate disease incidence rates. The high prevalence of prehospitalization antimicrobial drug use hampered accurate IPD surveillance. Failure to detect more antimicrobial-drug susceptible nonvaccine type infections as a result of prehospitalization antimicrobial drug use may have falsely elevated the proportion of disease covered by PCV13. The low prevalence of colonization among hospitalized children highlights the need for swab sample collection before in-hospital antimicrobial drug administration for accurate evaluation of colonization in unwell children. Because the study was conducted at 1 site, caution is required when extrapolating the results to the general population of Cambodia. These data provide a baseline against which to monitor effectiveness of vaccinating children with PCV13 in Cambodia.

**Technical Appendix.** Additional methods and results for study of pneumococcal infection among children before introduction of 13-valent pneumococcal conjugate vaccine, Cambodia.
