# Supplementary material for: Pneumococcal Infection among Children before Introduction of 13-Valent Pneumococcal Conjugate Vaccine, Cambodia
Source: Emerg Infect Dis. 2015 Nov;21(11):2080–3. doi: 10.3201/eid2111.150914 (PMC4622261; doi:10.3201/eid2111.150914)
Supplement: Supplementary file 1 — Technical Appendix. Additional methods and results for study of pneumococcal infection among children before introduction of 13-valent pneumococcal conjugate vaccine, Cambodia. [file 15-0914-Techapp-s1.pdf]

Article DOI: http://dx.doi.org/10.3201/eid2111.150914

Pneumococcal Infection among Children before Introduction of 13-Valent Pneumococcal Conjugate Vaccine, Cambodia

Technical Appendix

Study Site Details

Angkor Hospital for Children (AHC) is a non-governmental pediatric referral hospital in Siem Reap, Cambodia. The hospital serves the population of northern Cambodia: ≈300–500 children attend the out-patient department each day and there are around 4,000 medical admissions per year. In 2013, Cambodia had an estimated under-5 year mortality of 38 per 1000 births (*1*). The country has a tropical climate with monsoon rains between May and October each year.

Methods

Nasopharyngeal swab processing

Flocked nylon nasopharyngeal swabs (NPS; Medical Wire and Equipment, Corsham, UK) were collected in accordance with the updated WHO pneumococcal colonization protocol (*2*). The NPS tips was excised into 1mL STGG (skim milk, tryptone, glucose, glycerol medium; prepared in house) immediately after collection. The NPS-STGG specimens were stored in a cool box for <8 hours before definitive pre-culture storage at –80°C.

Thawed nasopharyngeal swab (NPS-STGG) specimens were vortexed and 100µL of each specimen was plated onto 5% sheep blood agar (prepared in-house) and incubated overnight in a candle jar. All morphologically discrete α-hemolytic colonies were sub-cultured and identified as *Streptococcus pneumoniae* by susceptibility to optochin disc (Oxoid, Basingstoke, UK), with confirmation by 10% bile solubility if optochin zone diameter was 7–14mm. All confirmed pneumococcal isolates were serotyped by latex

agglutination with Quellung confirmation of ambiguous results (*3*). A random selection of 21 phenotypically non-encapsulated non-typeable isolates were confirmed as pneumococci by bile solubility and absence of capsule swelling using Omniserum (SSI Diagnostica, Hillerød, Denmark).

Antimicrobial susceptibilities were determined for all isolates following 2013 CLSI guidelines (*4*). MICs (MIC) to benzylpenicillin and ceftriaxone were determined by the Etest method (bioMérieux, Marcy L'Etoile, France). Susceptibility to chloramphenicol, clindamycin, erythromycin, tetracycline, and trimethoprim-sulphamethoxazole was determined by disc diffusion. Non-susceptibility to penicillin and ceftriaxone was defined as an MIC of >0.06μg/mL and >1 μg/mL, respectively. Multi-drug resistance was defined as resistance (non-susceptibility for the β-lactam drugs) to three or more agents, with clindamycin/erythromycin and benzylpenicillin/ceftriaxone counting as a single agent (*5*).

All culture work was performed at the Angkor Hospital for Children / Cambodia Oxford Medical Research Unit microbiology laboratory which is located within the AHC campus. Internal quality control procedures were in place for all aspects of the laboratory work (media preparation, antimicrobial susceptibility testing, and serotyping).

Invasive pneumococcal disease data

At AHC, blood cultures are routinely taken in hospitalized children with fever or suspected invasive bacterial infection, with lumbar punctures also done in cases of suspected meningitis: between 1st January 2013 and 31st December 2014 11,238 blood cultures and 901 cerebrospinal fluid cultures were processed by the AHC microbiology laboratory. All invasive pneumococcal isolates (defined as *S. pneumoniae* isolated from blood, cerebrospinal fluid (CSF), pleural fluid and other usually sterile sites) cultured between 1st January 2013 and 31st December 2014 were retrieved from –80°C storage and sub-cultured onto 5% sheep blood agar for serotyping as described above. Antimicrobial susceptibilities for these isolates were extracted from the microbiology laboratory database.

Data analysis

Analyses were done using the R statistical package version 3.2 (R Foundation for Statistical Computing, Vienna, Austria). Pneumococcal serotypes were grouped in vaccine serotypes (PCV13: serotypes 1, 3, 4, 5, 6A, 6B, 7F, 9V, 14, 18C, 19F, 19A, 23F), non-vaccine serotypes (NVT: all other typeable pneumococci), and non-typeable pneumococci (NT). Colonization was a binary variable but children colonized by multiple pneumococci

could be included in more than serotype group (PCV13, NVT, or NT). Continuous variables were described using median and interquartile ranges; groups were compared using the Wilcoxon Rank Sum test. Proportions were compared using the Chi-squared or Fisher exact test, as appropriate. For these tests, two-tailed p-values of <0.05, were indicative of statistical significance. Multivariable logistic regression was used to assess factors potentially influencing pneumococcal colonization. Adjusted odds ratios (AOR) with 95% confidence intervals (CI) not including one were indicative of statistical significance.

Technical Appendix Table 1. Pneumococcal nasopharyngeal colonization and antibiotic exposure in 974 out-patient children, Angkor Hospital for Children, Siem Reap, Cambodia, 2014

Variable	Colonization, n (%)*			
All children (N)	Abx (48)	Possible Abx (283)	No Abx (643)	Total (974)
Colonized	25 (52.1)	168 (59.4)	408 (63.5)	601 (61.7)
Colonized children (N)	Abx (25)	Possible Abx (168)	No Abx (408)	Total (601)
PCV13 serotype(s)	20 (80.0)	111 (66.1)	267 (65.4)	398 (66.2)
NVT serotype(s)	4 (16.0)	56 (33.3)	130 (31.9)	190 (31.6)
NT isolate	2 (8.0)	11 (6.5)	38 (9.3)	51 (8.5)
MDR isolate(s)	20 (80.0)	127 (75.6)	280 (68.6)	427 (71.0)

*Colonization data are stratified by antimicrobial exposure in the month before collection of the nasopharyngeal swab sample. (Abx, definite antimicrobial exposure; Possible Abx, exposure to an unknown systemic medication; No Abx, no antimicrobial exposure. Children colonized by >1 pneumococcal serotype may be represented more than once within the colonized group (total number of colonized children = 601; total number of pneumococci isolated = 667).

Technical Appendix Table 2. Characteristics and WHO surveillance definition categorization of 1,009 hospitalized children enrolled into the colonization study at Angkor Hospital for Children, Siem Reap, Cambodia, August 2013–July 2014

	Age category, n (%)				
Characteristic	<2 mo	2–11 mo	12–59 mo	5–15 y	Total, n (col %)
Total enrolled*	77	358	444	130	1009
Male Sex (%)	49 (63.6)	212 (59.2)	238 (53.6)	71 (54.6)	570 (56.5)
Report antimicrobial use in preceding month, n (%)†	42 (54.5)	227/354 (64.1)	280/438 (63.9)	87/127 (68.5)	636/996 (63.9)
Household size, median (IQR)	6 (4 – 7)	5 (4 – 7)	5 (4 – 7)	5 (4 – 7)	5 (4 – 7)
Other children <5 years of age in household, n (%)	77 (100.0)	355/355 (100.0)	432/442 (97.7)	53/129 (41.1)	917/1003 (91.4)
Attendance at school or day care, n (%)	0/75 (0.0)	0/354 (100.0)	17/441 (3.9)	81/129 (62.8)	98/999 (9.8)
Surveillance category					
Severe pneumonia	61 (8.5)	290 (40.3)	304 (42.3)	64 (8.9)	719 (71.2)
Suspected meningitis	13 (8.6)	41 (27.0)	71 (46.6)	27 (17.8)	152 (15.1)
Pneumonia	0 (0.0)	20 (26.0)	46 (59.7)	11 (14.3)	77 (7.6)
Probable bacterial meningitis	1 (2.9)	5 (14.7)	16 (47.1)	12 (35.3)	34 (3.4)
Very severe disease	2 (8.7)	1 (4.3)	6 (26.1)	14 (60.9)	23 (2.3)
Confirmed bacterial meningitis	0 (0.0)	1 (25.0)	1 (25.0)	2 (50.0)	4 (0.4)

*Where there was missing data an alternate denominator is included in the affected cell.
†Includes definite and possible (unknown systemic medication) consumption in the community before hospitalization.

Technical Appendix Table 3. Pneumococcal nasopharyngeal colonization and antibiotic exposure in 1,008 hospitalized children, Angkor Hospital for Children, Siem Reap, Cambodia, 2014

Variable	Colonization, n (%)*			
All children (N)	**Abx (815)**	**Possible Abx (114)**	**No Abx (79)**	**Total (1008)**
Colonized	203 (24.9)	54 (47.7)	36 (45.6)	293 (29.1)
Colonized children (N)	**Abx (203)**	**Possible Abx (54)**	**No Abx (36)**	**Total (293)**
PCV13 serotype	155 (76.4)	34 (63.0)	26 (72.2)	215 (73.4)
NVT serotype	27 (13.3)	11 (20.4)	9 (25.0)	47 (16.0)
NT isolate	26 (12.8)	11 (20.4)	3 (8.3)	40 (13.7)
MDR isolate	165 (81.3)	39 (72.2)	30 (83.3)	234 (79.9)

*Colonization data are stratified by antimicrobial exposure in the month before the nasopharyngeal swab, including drugs administered in hospital before swabbing (Abx: definite antimicrobial exposure; Possible Abx: exposure to an unknown systemic medication; No Abx: no antimicrobial exposure). Children colonized by >1 pneumococcal serotype may be represented more than once within the colonized group (total number of colonized children = 293; total number of pneumococci isolated = 305).

Technical Appendix Table 4. Antimicrobial susceptibilities of colonizing and invasive pneumococci cultured from children attending Angkor Hospital for Children, Siem Reap, Cambodia, 2013–14

	PCV13 serotypes	NVT serotypes	NT isolates	All isolates
Colonization isolates	Resistant, n (%)*			
Out-patient				
Total isolates	418	197	52	667
Penicillin	383 (91.6)	96 (48.7)	44 (84.6)	523 (78.4)
Ceftriaxone	98 (23.4)	8 (4.1)	8 (15.4)	114 (17.1)
Clindamycin	199 (47.6)	38 (19.3)	10 (19.2)	247 (37.0)
Chloramphenicol	71 (17.0)	8 (4.1)	2 (3.8)	81 (12.1)
Co-trimoxazole	338 (80.9)	82 (41.6)	32 (61.5)	452 (67.8)
Erythromycin	265 (63.4)	42 (21.3)	12 (23.1)	319 (47.8)
Tetracycline	394 (94.3)	148 (75.1)	38 (73.1)	580 (87.0)
MDR	357 (85.4)	76 (38.6)	26 (50.0)	459 (68.8)
In-patient				
Total isolates	217	47	41	305
Penicillin	200 (92.2)	26 (55.3)	30 (73.2)	256 (83.9)
Ceftriaxone	42 (19.4)	4 (8.5)	3 (7.3)	49 (16.1)
Clindamycin	114 (52.5)	7 (14.9)	2 (4.9)	123 (40.3)
Chloramphenicol	46 (21.2)	4 (8.5)	4 (9.8)	54 (17.7)
Co-trimoxazole	204 (94.0)	29 (61.7)	26 (63.4)	259 (84.9)
Erythromycin	135 (62.2)	8 (17.0)	6 (14.6)	149 (48.9)
Tetracycline	207 (95.4)	39 (83.0)	39 (95.1)	285 (93.4)
MDR	197 (90.8)	21 (44.7)	24 (58.5)	242 (79.3)
Invasive pneumococcal isolates				
Total isolates	38	3	2	43
Penicillin	20 (52.6)	1 (33.3)	1 (50.0)	22 (51.2)
Ceftriaxone	0 (0)	0 (0)	0 (0)	0 (0)
Clindamycin	11 (28.9)	0 (0)	0 (0)	11 (25.6)
Chloramphenicol	16 (42.1)	1 (33.3)	0 (0)	17 (39.5)
Co-trimoxazole	32 (84.2)	0 (0)	2 (100)	34 (79.1)
Erythromycin	12 (31.6)	0 (0)	0 (0)	12 (27.9)
Tetracycline	36 (94.7)	3 (100)	2 (100)	41 (95.3)
MDR	22 (57.9)	1 (33.3)	1 (50.0)	24 (55.8)

*Antimicrobial susceptibilities were determined following 2013 CLSI guidelines (*4*). Resistance was defined as disk test "resistant" except for the β-lactam drugs: benzylpenicillin MICs of >0.06 μg/mL were classified as "resistant"; ceftriaxone MICs of >1 μg/mL were classified as "resistant." Multidrug resistance (MDR) was defined as resistance to three or more agents, with clindamycin/erythromycin and benzylpenicillin/ceftriaxone counting as a single agent (*5*).

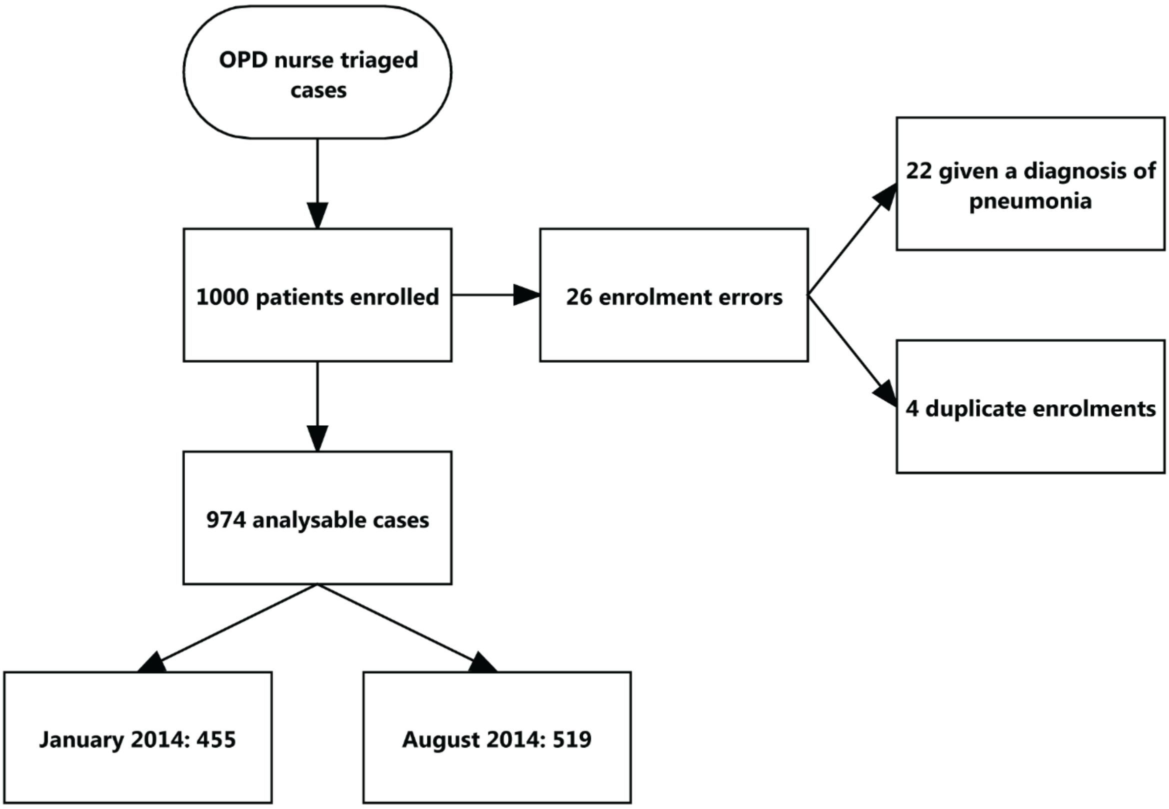

Technical Appendix Figure 1. Study flowchart for the out-patient pneumococcal colonization surveys at Angkor Hospital for Children, Siem Reap, Cambodia, January and August 2014.

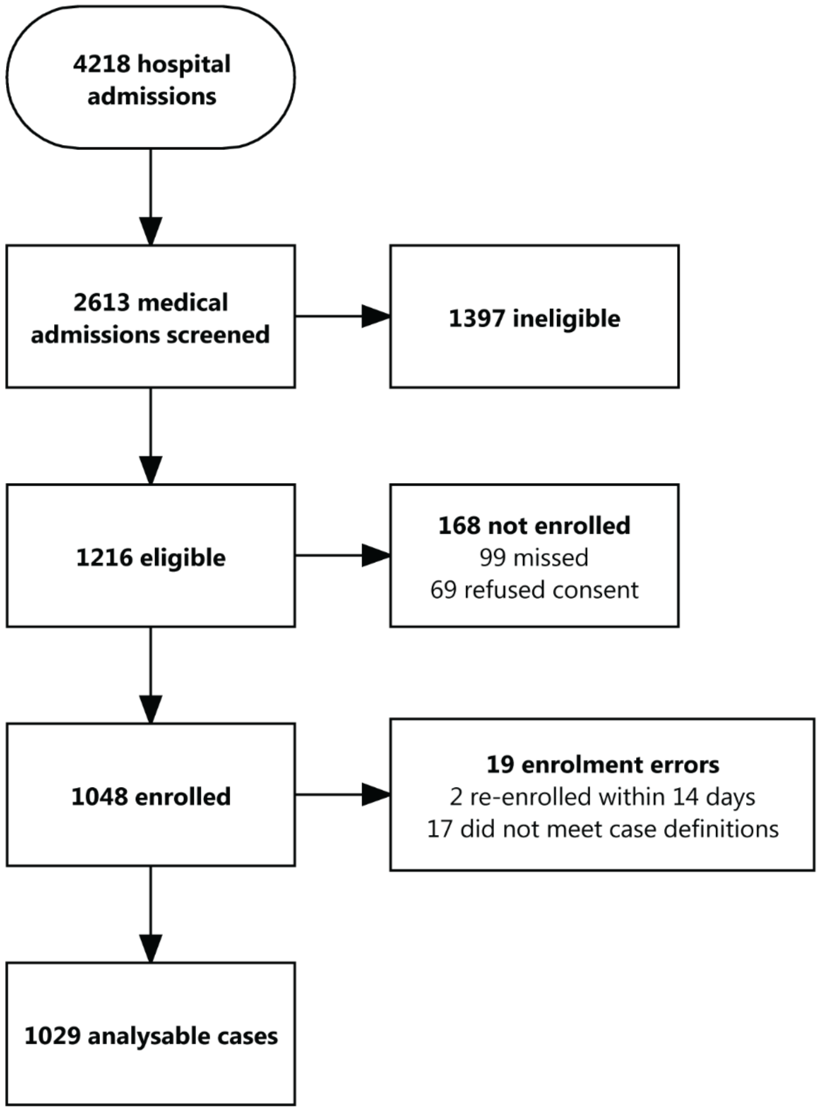

Technical Appendix Figure 2. Study flowchart for the hospitalized patient pneumococcal colonization study at Angkor Hospital for Children, Siem Reap, Cambodia, August 2013–July 2014.

References

1. Alliance GAVI. Cambodia [cited 2015 Apr 25]. http://www.gavi.org/country/cambodia/

2. Satzke C, Turner P, Virolainen-Julkunen A, Adrian PV, Antonio M, Hare KM, et al. Standard method for detecting upper respiratory carriage of *Streptococcus pneumoniae*: updated recommendations from the World Health Organization Pneumococcal Carriage Working Group. Vaccine. 2013;32:165–79. PubMed http://dx.doi.org/10.1016/j.vaccine.2013.08.062

3. Turner P, Turner C, Jankhot A, Helen N, Lee SJ, Day NP, et al. A longitudinal study of *Streptococcus pneumoniae* carriage in a cohort of infants and their mothers on the Thailand–Myanmar border. PLoS ONE. 2012;7:e38271. PubMed http://dx.doi.org/10.1371/journal.pone.0038271

4. Clinical and Laboratory Standards Institute. Performance standards for antimicrobial susceptibility testing. 17th informational supplement. CLSI document M100–S23. Wayne (PA): The Institute; 2013.

5. von Gottberg A, de Gouveia L, Tempia S, Quan V, Meiring S, von Mollendorf C, et al. Effects of vaccination on invasive pneumococcal disease in South Africa. N Engl J Med. 2014;371:1889–99. PubMed http://dx.doi.org/10.1056/NEJMoa1401914
